# Modulating Anti-MicroRNA-21 Activity and Specificity Using Oligonucleotide Derivatives and Length Optimization

**DOI:** 10.5402/2012/407154

**Published:** 2012-02-07

**Authors:** Andrés Muñoz-Alarcón, Peter Guterstam, Cristian Romero, Mark A. Behlke, Kim A. Lennox, Jesper Wengel, Samir EL Andaloussi, Ülo Langel

**Affiliations:** ^1^Department of Neurochemistry, Stockholm University, Svante Arrhenius väg 21A, 106 92 Stockholm, Sweden; ^2^GE Healthcare Bio-Sciences, Björkgatan 30, 751 84 Uppsala, Sweden; ^3^Integrated DNA Technologies, 1710 Commercial Park, Coralville, IA 52241, USA; ^4^Nucleic Acid Center, Department of Physics and Chemistry, University of Southern Denmark, 5230 Odense M, Denmark; ^5^Department of Laboratory Medicine, Karolinska Institute, Hälsovägen 7, 141 86 Huddinge, Sweden

## Abstract

MicroRNAs are short, endogenous RNAs that direct posttranscriptional regulation of gene expression vital for many developmental and cellular functions. Implicated in the pathogenesis of several human diseases, this group of RNAs provides interesting targets for therapeutic intervention. Anti-microRNA oligonucleotides constitute a class of synthetic antisense oligonucleotides used to interfere with microRNAs. In this study, we investigate the effects of chemical modifications and truncations on activity and specificity of anti-microRNA oligonucleotides targeting microRNA-21. We observed an increased activity but reduced specificity when incorporating locked nucleic acid monomers, whereas the opposite was observed when introducing unlocked nucleic acid monomers. Our data suggest that phosphorothioate anti-microRNA oligonucleotides yield a greater activity than their phosphodiester counterparts and that a moderate truncation of the anti-microRNA oligonucleotide improves specificity without significantly losing activity. These results provide useful insights for design of anti-microRNA oligonucleotides to achieve both high activity as well as efficient mismatch discrimination.

## 1. Introduction

Originally identified in *Caenorhabditis elegans* and subsequently established in a number of additional organisms including mammalian cells [1–3], RNA interference (RNAi) is a posttranscriptional gene-silencing process targeting single-stranded RNA sequences. Whereas several classes of RNAi effectors have been identified, siRNAs and microRNAs (miRNAs) are the best characterized.

 miRNAs interact with transcript sequences possessing partial or full complementarity, promoting gene repression of the targeted transcripts. Dysregulation of miRNAs has been implicated in human developmental disorders and diseases, including several forms of cancer [4–6]. Manipulation of miRNAs, utilizing synthetic, chemically modified oligonucleotides (ONs) targeting select miRNAs, presents an approach to both elucidate the role of miRNA dysregulation in human disease and discover novel therapies for many pathological conditions. miRNAs have previously been shown to possess the ability to regulate multiple functionally related mRNAs, such as sets of metabolic genes [7, 8], a powerful feature that may enable miRNA-based therapeutics to circumvent redundant mechanisms that might otherwise bypass single inhibited targets.

 miRNA-21 (miR21) is potentially a very interesting target for future therapeutic applications. It has widespread regulatory functions and has been implicated in a variety of diseases, including cancer and heart disease [9, 10]. It is overexpressed in many forms of human cancers and has been shown to be an important regulator of many tumor suppressors [9, 11–14].

 ON technologies provide specific and powerful means for targeting biologically active nucleic acids for inhibition. Unmodified DNA ONs have previously been shown to be less effective as inhibitors due to chemical instability *in vivo* [15]. Subsequently, chemical modification of nucleotide monomers has been explored as a means to modulate affinity and bioactivity of nucleic acids [16–19]. The most important property of chemically modified ONs is specificity and high binding affinity to RNA. A number of chemically altered nucleotides with increased binding affinity have been synthesized. Optimally, an anti-miRNA oligonucleotide (AMO) should be stable *in vivo*, hybridize efficiently and specifically with the target miRNA, be nontoxic and nuclease resistant. The 2′-O-methyl RNA (2′OMe RNA) possesses increased nuclease resistance and improved base-pairing affinity as compared to both DNA and RNA. Incorporation of locked nucleic acid (LNA) monomers confers a well-documented affinity enhancement to complementary sequences [20–23]. Unlocked nucleic acid (UNA) is an acyclic RNA analogue that has been shown to destabilize duplexes formed between UNA-modified RNA strands and complementary DNA or RNA. As such, UNA monomers are able to modulate base-pairing specificity and duplex stability [24]. Phosphorothioates are a variant of normal DNA in which one of the nonbridging oxygens in the phosphate backbone is replaced by sulphur. This chemical modification has been shown to drastically improve nuclease resistance as well as the ability to cross the lipid bilayer of the cell membrane [25].

 In the present study a set of AMOs complementary to miR21 were synthesized (Table 1). The AMOs differed in length and chemical composition, such as the amount and type of synthetic nucleotide monomers incorporated. Corresponding AMOs with two mismatches were utilized to assess mismatch discrimination. 

The main scope of our study was to assess the effects on AMO specificity and activity conveyed by synthetic nucleotides with different chemical modifications and ON length. 

## 2. Materials and Methods

### 2.1. Synthesis of Oligonucleotides

The phosphorothioate 2′OMe RNA AMOs were synthesized on a ÄKTA oligopilot plus 10 synthesizer (GE Healthcare) and purified as previously described [26]. All AMOs, phosphorothioate and phosphodiester, with LNA or UNA content were obtained from RiboTask. All phosphorothioate AMOs are denoted with PS; ONs lacking this denotation are phosphodiester AMOs.

### 2.2. Cell Culture

HeLa cells were grown in DMEM (Dulbecco's modified Eagle's medium) with glutamax supplemented with 0.1 mM nonessential amino acids, 1.0 mM sodium pyruvate, 10% fetal bovine serum (FBS), 100 units/ml penicillin, and 100 mg/ml streptomycin. Cells were cultured at 37°C in a 5% CO_2_ atmosphere. All media and chemicals were purchased from Invitrogen.

### 2.3. Anti-MiRNA Assay

To evaluate the AMOs we used a psiCHECK-2 vector (Promega) with a miR21-binding site cloned into the 3′ UTR of the *Renilla* luciferase gene. In addition to the *Renilla* luciferase gene, the psiCHECK-2 vector also contains a constitutively active firefly luciferase gene used for normalization of protein content. In the absence of an AMO to provide effective miRNA inhibition, endogenous miR21 blocks *Renilla* luciferase expression. Thus the level of *Renilla* luciferase expression will be a measurement of the AMO activity.

 HeLa cells (600 000 cells) were plated in a 60 mm dish. 24 hours later 2 *μ*g PsiCHECK-2 plasmid was transfected with 4 *μ*l of Lipofectamine 2000. The transfection mix was added to the cells growing in 4 ml of medium. 24 hours later cells were trypsinized and counted. 60 000 cells/well were replated in 24 well plates (Sigma-Aldrich) with 1 ml DMEM + 10% FBS. 24 hours after plasmid transfection, anti-miR21 ONs were transfected using Lipofectamine 2000, in a total volume of 500 *μ*L. Anti-miR21 ONs were transfected in triplicates at 100 nM, 50 nM, 25 nM, or 10 nM. After 6 hours, 500 *μ*L DMEM + 10% FBS was added to each well. 24 hours after anti-miR21 transfection, cells were lysed and luciferase activity of 20 *μ*L of cell lysate was measured in RLUs (relative luminescence units) on a GloMax 96 microplate Luminometer (GloMax) using the Dual-Luciferase Reporter Assay System (Promega). The results are presented as normalized luciferase activity, which corresponds to the increase of luciferase activity compared to untreated cells. Experiments were performed at least three times in triplicate.

### 2.4. Statistical Analyses

All results are mean ±SEM for at least two independent experiments performed in triplicate. Statistics were calculated using two-tailed paired *t*-tests (***P < 0.001, **P < 0.01, *P < 0.05).

## 3. Results

### 3.1. Design of AMOs

To assess and compare the impact on AMO activity mediated by different synthetic ONs and ON length we designed a set of ONs targeting miR21 using different ON chemistries and lengths (Table 1). ON length ranged from 9 nucleotides to 22 nucleotides. Synthesized ONs were 2′OMe RNA, LNA/2′OMe RNA mixmers with or without phosphorothioate backbone, and LNA/2′OMe/UNA mixmers (Table 1), all targeting miR21. Content of UNA and LNA is given as percent, any remaining nucleotides are 2′OMe ribonucleotides.

 We designed LNA/2′OMe/UNA mixmers to investigate whether it was possible to use UNA to modulate any potential negative impact on mismatch discrimination mediated by LNA. Such effects of LNA on specificity were previously reported for splice-switching ONs (SSOs) with LNA content [26]. In order to evaluate effects on specificity, we synthesized corresponding ONs with two mismatches (2MM) to the targeted miR21. Using the Dual-Luciferase Reporter Assay System (Promega), an increase in luciferase activity normalized against untreated cells was used as indication of the efficiency of the AMOs to hybridize and interfere with miR21 binding to the miR21-binding site in the psiCHECK-2 vector. Differences in activity between wild-type (wt) AMOs and their corresponding control ONs with two bases mismatched for the target sequence was used as an indication of specificity. All mismatched bases were mismatched against the bases 9 and 11 in the target miR21 sequence.

### 3.2. Oligonucleotide Length Optimization

**Figure 1:**
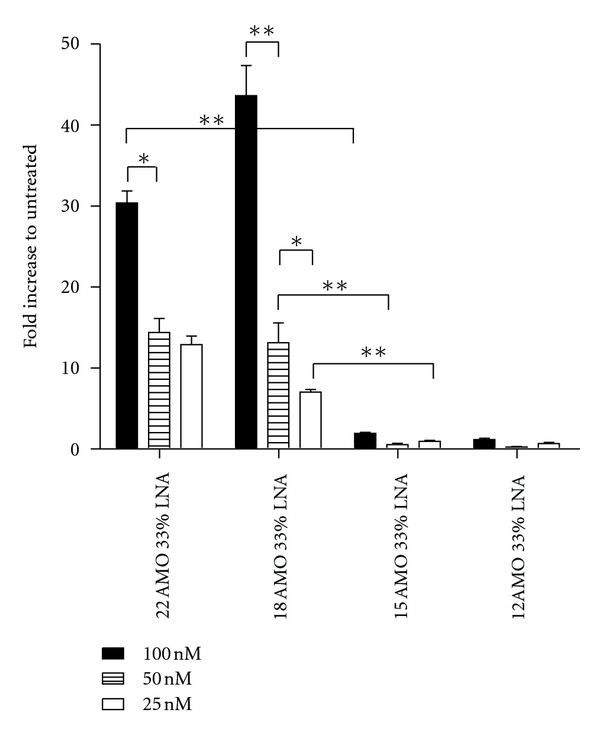
Oligonucleotide length effects on AMO activity. Fold increase of luciferase activity after wild-type AMO treatment at three different concentrations, 100 nM, 50 nM, and 25 nM. Five different lengths were used, a 22-, 18-, 15-, 12-, and 9-mer (data not shown) ONs. All AMOs had a phosphodiester (PO) backbone.

**Figure 2:**
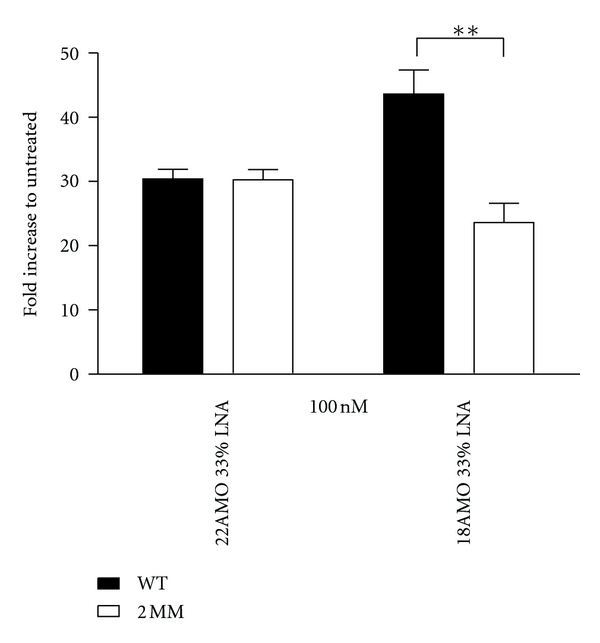
Influence of oligonucleotide length on AMO specificity. Fold increase of luciferase activity posttreatment at 100 nM concentration with a 32% LNA 22-mer AMO and 33% LNA 18-mer, both with phosphodiester backbone. The 18-mer AMO has a comparative activity to that of the 22-mer AMO but the AMO with two mismatches has lower activity than the corresponding 22-mer with two mismatches, indicating better sequence specificity for target miR21.

As previous studies have shown that a moderate reduction of ON length not necessarily compromises the activity [27], we tested a range of AMOs of various lengths to see if truncation had similar effects on the activities of the AMOs. The activity was not significantly different between the 22-nucleotide-long ON and the 18-nucleotide-long ON. However, at a length of 15 nucleotides or shorter the activity was all but abolished completely (Figure 1). The 9-mers did not display any activity and were thus omitted from the figures. Concomitantly, the difference in activity between wt and 2MM AMOs was significant for the AMOs with a length of 18 nucleotides as opposed to the full 22-nucleotide-long AMOs (Figure 2). Thus the 18-mers displayed a greater discrimination against mismatches, and therefore a higher specificity, than the 22-mers. Additionally, the activity of the 18-mer was significantly lower at lower concentrations, whereas the dose dependency of the activity of the 22-mer was not as statistically acute. 

### 3.3. Oligonucleotide Modification Effects on AMO Activity and Specificity

**Figure 3:**
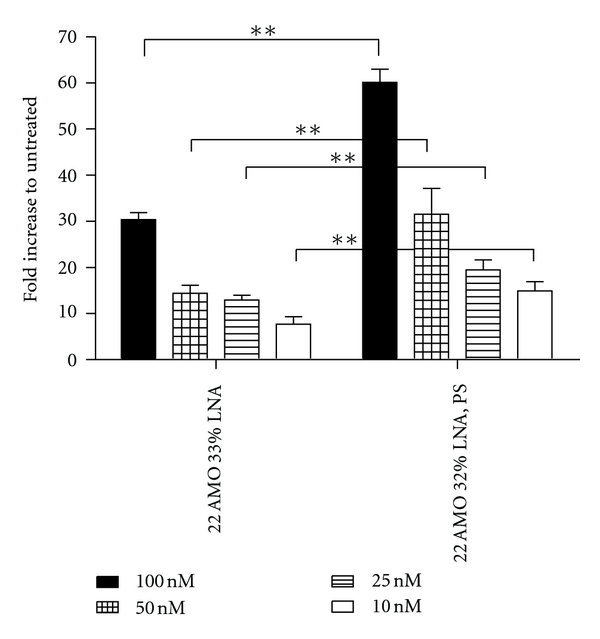
Influence of phosphorothioates on AMO activity. Fold increase of luciferase activity after wild-type AMO treatment at 100 nM, 50 nM, 25 nM, and 10 nM concentrations. The AMO activity was at all concentrations significantly greater for the phosphorothioate (PS) ONs than the activity of the phosphodiester ONs.

We investigated the effects on AMO activity mediated by phosphodiester chemistry by comparing the activity of phosphorothioate LNA/2′OMe RNA mixmers to that of phosphodiester LNA/2′OMe RNA mixmers at four different concentrations, 100 nM, 50 nM, 25 nM, and 10 nM. The activity for the LNA/2′OMe RNA mixmers with a phosphorothioate backbone was significantly greater than those with a normal phosphodiester backbone at all tested concentrations, respectively (Figure 3). 

**Figure 4:**
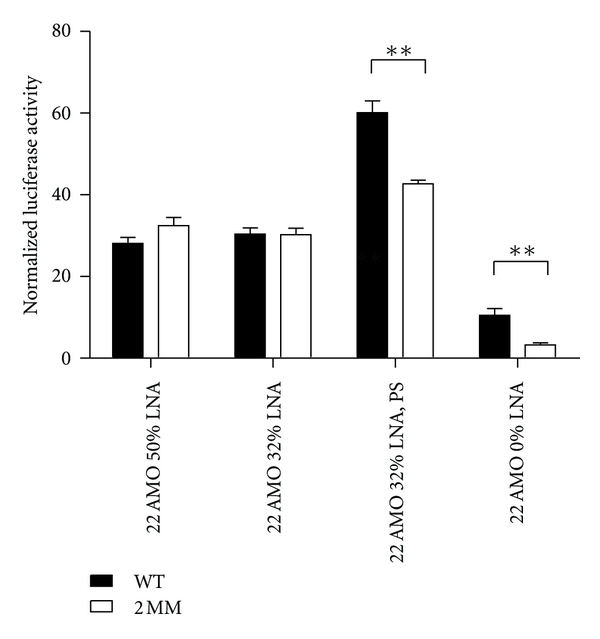
LNA and phosphorohioate effects on AMO activity and specificity. Fold increase of luciferase activity after AMO treatment at 100 nM concentrations. The amount of LNA monomers incorporated in the oligonucleotides used varied between 0% and 50%. No significant disparity in activity was seen between 32% and 50% LNA content. The activity of AMOs with 32% and 50% LNA was significantly greater than with 0% LNA. Phosphorothioate AMO with 32% LNA and AMO absent of LNA both showed a significant difference in activity between wildtype and mismatched construct.

 We also investigated the effects on AMO interaction with miR21 mediated through introduction of LNA into AMOs. LNA-enhanced activity is supported by the well-characterized high binding affinity for LNA residues [18]. The addition of LNA monomers resulted in an increase in activity but at the cost of reduced specificity, except in the case of the phosphorothioate LNA/2′OMe mixmer (Figure 4). The activity of the AMO with 32% LNA was not significantly different from that of the 50% LNA AMO (Figure 4). 

**Figure 5:**
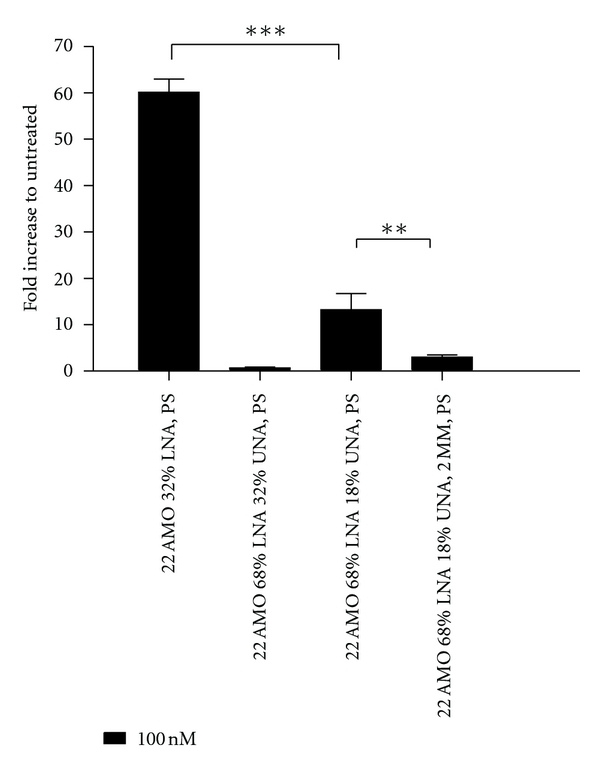
Influence of UNA monomers on AMO activity. Fold increase of luciferase activity measured after treatment with 100 nM AMO, comparing the 22AMO with phosphorothioate backbone and 32% LNA with the 22AMO with LNA/UNA. UNA content improved mismatch discrimination.

 The LNA/2′OMe/UNA mixmers with a UNA content of 32% displayed virtually no activity, whereas significant activity was obtained using similar mixmers with 18% UNA. High UNA content appeared to all but totally abolish the AMO activity but lower UNA content exhibits mismatch discrimination (Figure 5).

## 4. Discussion

MiRNAs, one of several types of RNAi effectors, provide possible therapeutic targets to combat human diseases and developmental disorders. They also provide a platform to further the understanding of the mechanisms of RNA interference. AMOs can be used for this purpose as either a therapeutic agent [28, 29] or as a tool to study miRNAs. Some of the key properties of an ideal AMO for therapeutic purposes would be serum stability, a high activity at low concentrations, and a high specificity for its target sequence. 

 In the present study we used a set of synthesized AMOs of different lengths and different nucleotide chemistries to target miR21, a commonly expressed miRNA, and an important regulator of cancer-cell survival [11–14]. Our results suggest that while introducing LNA monomers into the AMOs improves the activity it also reduces the specificity (Figure 4), which is important to consider when designing AMOs. This is consistent with observations done in experiments with splice-switching oligonucleotides (SSOs) and antisense LNA mixmers [13, 26, 30]. 

 Changing the AMO backbone chemistry provides yet another means to modulate activity and specificity. Our results indicate that LNA mixmer AMOs with a phosphorothioate backbone yield a much greater activity than with a phosphodiester backbone (Figure 3). This could in part be explained by a better cellular binding and uptake as well as better serum stability of the phosphorothioate AMO compared to AMOs with a phosphodiester chemistry [31]. Phosphorothioate AMOs also displayed good mismatch discrimination despite a 32% LNA content (Figure 4). Phosphorothioate linkages lower binding affinity by ~0.43°C/linkage which can increase specificity [32]. However, PS linkages may cause toxicity through nonspecific interactions with proteins [33]. 

 Besides introducing a phosphorothioate backbone chemistry, the negative impact on mismatch discrimination conveyed by the LNA monomers appears to be possible to counteract through a shortening of the ON. Our results show that it is possible to shorten the AMO while retaining activity and that the 18-mer displays improved mismatch discrimination compared to the full-length 22-mer AMO (Figure 2). This is in accordance with previous studies of antisense ON length effects on activity, showing the possibility of retaining a high activity with shortened ONs [27]. In addition, recent studies show that the specificity of particularly higher potency compounds benefit from use of lower dosages [34].

 UNA has previously been reported to have duplex-destabilizing properties [24]. Consequently, LNA/UNA mixmers were used to explore the possibility to modulate the duplex-stabilizing property of LNA mixmers with the destabilizing property of the UNA monomers. This was done to assess whether introducing UNA monomers could reduce the negative impact of LNA on mismatch discrimination. However, the 68% LNA and 32% UNA AMO used in the study displayed virtually no activity (Figure 5) making a specificity comparison impossible. It appears the LNA to UNA ratio was not balanced enough to prevent the detrimental effect of UNA monomers on duplex stability to all but completely abolish the AMO activity. A significant albeit low activity was obtained by performing the experiment using mixmers with 18% UNA content instead. Moreover, those mixmers displayed a significant mismatch discrimination (Figure 5), whereas the LNA/2′OMe mixmer completely lacking UNA did not (Figure 4). 

 In summary, our results suggest that introducing LNA monomers to 2′-OMe RNA ONs enhances AMO activity but mismatch discrimination is adversely affected. An increased activity can also be obtained through chemical modification of the ON backbone as a phosphorothioate ON with LNA monomers introduced. AMOs which are 18-nucleotide-long LNA/2′-OMe RNA mixmers display an activity comparable to full-length 22-mers but with higher specificity. This could be of importance during ON design as designing shorter AMOs could compensate potential toxicity due to low mismatch discrimination. The negative effect of LNA on specificity to target miRNAs has to be considered when utilizing LNA monomers in mixmers for targeting miRNAs. Although our results showed no significant difference in specificity between AMOs with 32% and 50% LNA (Figure 4) it might be possible to improve the specificity through tuning of the proportion of LNA content. The addition of UNA monomers may contribute to improved mismatch discrimination yet abolishes AMO activity if the content is high.

## 5. Conclusions

Our observations are generally in accordance with previous studies. AMOs modified with LNA and/or phosphorothioates were very potent *in vitro* using miR21 reporter assay systems yet we observed a deleterious effect on specificity caused by the introduction of LNA monomers. UNA-modified AMOs displayed a drastically reduced potency but conversely appeared to improve the specificity. Furthermore, reducing the length of the AMO from 22 nucleotides to 18 nucleotides improves mismatch discrimination while retaining activity. This highlights the possibility to balance the potent activity-enhancing modifications such as LNA with specificity-improving modifications such as truncation and moderate incorporation of UNA monomers. These data should aid researchers in rational design of AMO and further investigations of modification schemes suited to their individual requirements.

## Figures and Tables

**Table 1:** Oligonucleotides used in this study.The anti-miR21 ONs used in this study, ranging from 9 to 22 nucleotides in length. The ONs are 2′OMe RNA, LNA/2′OMe RNA mixmers with or without phosphorothioate backbone, and LNA/2′OMe/UNA mixmers. Corresponding ONs with 2MMs were synthesized. Mismatched bases are mismatched against the bases 9 and 11 in the target miR21 sequence. Phosphorothioate AMOs are denoted with PS, ONs lacking this denotation are phosphodiester AMOs. Capital letters represent 2′OMe ribonucleotides. Lowercase letters indicate LNA monomers, whereas underlined letters represent UNA monomers. Italic letters mark mismatches and bold letters phosphorothioates.

Sequence	Name	Chemistry	Mismatches	LNA-mismatches
3′- AGU UGU AGU CAG ACU AUU CGA U-5′	Original miR21 sequence		0	0
5′-UCA ACA UCA GUC UGA UAA GCU A-3′	22AMO	0% LNA	0	0
5′-UCA ACA UC*U* G*A*C UGA UAA GCU A-3′	22AMO_2MM	0% LNA	2	0
5′- UcA aCa UcA gUc UgA uAa GcU a-3′	22AMO_50%	50% LNA	0	0
5′- UcA aCa Uc***U*** g***A***c UgA uAa GcU a-3′	22AMO_50%_2MM	50% LNA	2	0
5′- UcA AcA UcA GuC UgA UaA GcU A-3′	22AMO_32%	32% LNA	0	0
5′- UcA AcA Uc***U*** G***a***C UgA UaA GcU A-3′	22AMO_32%_2MM	32% LNA	2	1
5′-** UcA AcA UcA GuC UgA UaA GcU A**-3′	22AMO_32%_PS	PS 32% LNA	0	0
5′-** UcA AcA Uc*U* G*a*C UgA UaA GcU A**-3′	22AMO_32%_2MM_PS	PS 32% LNA	2	1
5′- AcA UcA GuC UgA UaA GcU -3′	18AMO_33%	33% LNA	0	0
5′- AcA Uc*U* G*a*C UgA UaA GcU -3′	18AMO_33%_2MM	33% LNA	2	1
5′- AcA UcA GuC UgA UaA -3′	15AMO_33%	33% LNA	0	0
5′- AcA Uc***U*** G***a***C UgA UaA -3′	15AMO_33%_2MM	33% LNA	2	1
5′- AcA UcA GuC UgA -3′	12AMO_33%	33% LNA	0	0
5′- AcA Uc***U*** G***a***C UgA -3′	12AMO_33%_2MM	33% LNA	2	1
5′- UcA GuC UgA -3′	9AMO_33%	33% LNA	0	0
5′- Uc***U*** G***a***C UgA -3′	9AMO_33%_2MM	33% LNA	2	1
5′-**UcA AcA UcA GuC UgA UaA GcU A**-3′	22AMO_68%_PS	PS 68% LNA,32%UNA	0	0
5′-**UcA AcA Uc*U* G*a *CUgA UaA GcU A**-3′	22AMO_68%_2MM_PS	PS 68% LNA,32%UNA	2	1
5′-** uCa aca uCa guc uGa uaa gCu a**-3′	22AMO_68%_PS	PS 68% LNA,18%UNA	0	0
5′-** uCa aca uC*u* g*A*c uGa uaa gCu a**-3′	22AMO_68%_2MM_PS	PS 68% LNA,18%UNA	2	1
